# Safety, Effectiveness, and Hemodynamic Performance of the Bovine Pericardium Organic Valvular Bioprosthesis

**DOI:** 10.21470/1678-9741-2023-0015

**Published:** 2023-08-07

**Authors:** Álvaro Machado Rösler, Fernando Antonio Lucchese, Pablo Maria Alberto Pomerantzeff, Luiz Carlos Santana Passos

**Affiliations:** 1 Department of Cardiovascular Surgery, Hospital São Francisco, Santa Casa de Misericórdia de Porto Alegre, Porto Alegre, Rio Grande do Sul, Brazil; 2 Department of Cardiovascular Surgery, Instituto do Coração, Hospital das Clínicas, Faculdade de Medicina, Universidade de São Paulo (INCOR-HCFMUSP), São Paulo, São Paulo, Brazil; 3 Department of Cardiovascular Surgery, Hospital Ana Nery, Salvador, Bahia, Brazil

**Keywords:** Aortic Valve, Bioprosthesis, Animals, Heart Valve Prosthesis, Hemodynamics, Prosthesis Design, Treatment Outcomes

## Abstract

**Objective:**

To assess actual data on the safety, effectiveness, and hemodynamic performance of Bovine Pericardium Organic Valvular Bioprosthesis (BVP).

**Methods:**

The BIOPRO Trial is an observational, retrospective, non-comparative, non-randomized, and multicenter study. We collected data from 903 patients with symptomatic, moderate, or severe valve disease who underwent BVP implants in the timeframe from 2013 to 2020 at three Brazilian institutions. Death, valve-related adverse events (AEs), functional recovery, and hemodynamic performance were evaluated at the hospital, at discharge, and six months and one year later. Primary analysis compared late (> 30 days after implant) linearized rates of valve-related AEs, such as thromboembolism, valve thrombosis, major hemorrhage, major paravalvular leak, and endocarditis, following objective performance criteria (OPC). Analysis was performed to include at least 400 valve-years for each valve position (aortic and mitral) for complete comparisons to OPC. Kaplan-Meier survival and major adverse cardiovascular and cerebrovascular event analyses were also performed.

**Results:**

This retrospective study analyzed follow-up data collected from 903 patients (834.2 late patient-years) who have undergone surgery for 455 isolated aortic valve replacement (50.4%), 382 isolated mitral valve replacement (42.3%), and 66 combined valve replacement or other intervention (7.3%). The linearized rates of valve-related AEs were < 2 × OPC. One-year survival rates were 95.1% and 92.7% for aortic and mitral valve replacement, respectively. This study demonstrated an improvement in the New York Heart Association classification from baseline and hemodynamic performance within an expected range.

**Conclusion:**

According to this analysis, BVP meets world standards for safety and clinical efficacy.

## INTRODUCTION

Heart valve is a structure in the circulatory system that allows blood to flow only in one direction and is closely associated with hemodynamic function but can be susceptible to serious pathologies resulting from congenital malformations, rheumatic diseases, infectious diseases, arthritis, and structural degeneration caused primarily by calcification^[1]^. When a natural heart valve becomes defective, it can result in stenosis or regurgitation. These problems can occur on just one or more than one valve.

Treatment options for heart valve disease include medication, surgical repair, or replacement. Based on the material used in cardiac valve prostheses, there are two major categories: (a) bioprosthesis or biological valves made basically of animal tissue, such as bovine pericardium, and (b) mechanical valves made of synthetic materials^[2]^.

Although biological valves are practically non-thrombogenic^[1,2,3,4,5]^, they are less durable, mainly due to structural degeneration induced by the calcification process. As a general rule, as the patient ages and/or the risk of thrombogenicity increases, biological valves are recommended for use^[5]^.

The Bovine Pericardium Organic Valvular Bioprosthesis (BVP) (Braile Biomédica®) was introduced to clinical use in 1977. Based on results of several studies, this valve is widely and successfully used to treat valve diseases. BVP exhibits excellent hemodynamics and a minimal rate of valve-related adverse events (AEs). In this article, we presented actual results from a retrospective trial investigating this prosthesis in a cohort of patients undergoing surgical valve replacement.

## METHODS

### Study Design

The BIOPRO Trial is a retrospective, non-randomized, multicenter study designed to perform an update on the safety and effectiveness evaluations of the BVP (Braile Biomédica®). This study complies with the Declaration of Helsinki and was performed according to the local ethics committee’s approval (CIP identification #191031), following the International Organization for Standardization [ISO] 14155:2011^[6]^ and ISO 5840-2 recommendations^[7]^. The trial was performed at three cardiology reference centers in Brazil: 1) Hospital Ana Nery (State of Bahia); 2) Hospital São Francisco (State of Rio Grande do Sul), and 3) Instituto do Coração, Universidade de São Paulo (State of São Paulo).

Patients who have received heart valves implanted at these institutions tend to remain under follow-up at specialized ambulatory. Data were obtained from patients’ records and examinations performed during the first year after valve surgery, including baseline clinical data, procedure information, mortality, AEs, and New York Heart Association (NYHA) class.

Multiple patient records were tracked on electronic Case Report Forms (e-CRFs) provided by Braile Biomédica®. A professional from Core Lab (Le Bihan Cardiologia e Anestesia S/S Ltda) validated the echocardiographic data about the device’s hemodynamic performance.

### Device and Procedure

The BVP (supplementary material S1) is a stented, pericardial tissue valve that is indicated for replacement of aortic and mitral valves. The prosthesis size is available in the following diameters: 19, 21, 23, 25, 27, 29, 31, and 33 mm. In this study, all patients underwent traditional surgery with standard operative techniques for valve implantation. Interventions included cardiopulmonary bypass with moderate hypothermia, aortic cannulation, and single or double venous cannulation for patients who had mitral valve involvement.

### Study Population

Nine hundred and three patients with native valve or prosthetic aortic and/or mitral replacement were included in the BIOPRO Trial analysis group. The patients who have received biological heart valves were followed up to one year after the procedure, meeting the requirements of the ISO guidance. All operated patients available for follow-up underwent routine clinical examinations such as echocardiography and blood biochemistry.

This study followed “real life” practices for surgical valve replacement and standard of care at participating sites. Inclusion and exclusion criteria are described in S2. The inclusion criteria were based on the European Society of Cardiology/European Association for Cardio-Thoracic Surgery Guidelines to extrapolate the results to the European Union population^[8]^.

### Baseline, Perioperative, and Follow-up Evaluations

Baseline evaluation included a collection of demographic characteristics, laboratory data, comorbid conditions, rhythm on electrocardiogram (ECG), previous interventions, and echocardiogram evaluation with assessment of NYHA functional status.

Perioperative evaluation included a data collection of additional required procedures or interventions, device failure or malfunction, mortality (related or not to the valve), and complications (related or not to the valve).

Patients were scheduled for follow-up at hospital discharge (up to 30 days), three to six months, and one year later. These data included an assessment of NYHA classification, ECG, and linearized rates of AEs.

### Study Endpoints

Safety endpoints include freedom from specific complications, including but not limited to thromboembolism, valve thrombosis, major hemorrhage, major paravalvular leak, and endocarditis, according to the ISO 5840-2 – objective performance criteria (OPC)^[9]^. The linearized rates (%/patient-year) of late AEs obtained by late events occurring after the 31^st^ day of implantation and “freedom from the event” at one-year based on Kaplan-Meier analysis are provided based on all reported events.

### Statistical Analysis

Patient data captured on e-CRF were collected in a worksheet and/ or directly from health professionals based on consultations with patients at the clinic for pertinent statistical analysis, considering that the significance level was 0.05.

Descriptive statistics are used to report clinical characteristics, qualitative variables were reported by absolute frequency (n) and relative frequency (percentage), and continuous variables are used to obtain mean and standard deviation. Linearized rates of late AEs are calculated as the total number of late events (those occurring > 30 days after implant) divided by the total follow-up time, expressed as a percentage^[10]^.

## RESULTS

Study population consisted of typical individuals who needed replacement of their native or prosthetic valve, according to the instructions for use, and all patients following “real life” practices and standard of care at three participating Brazilian institutions. Analysis of effectiveness was based on the 903 patients that received BVP for 839 total patient-years.

Based on Table 1, nearly 19.2% of patients had undergone previous cardiac surgery, 17.0% had one surgery, and 2.1% had two previous surgeries. Mean age in the study group (59.4±14.3 years) and percentage of patients in NYHA functional class II (31.4%) and III (32.7%) were higher than in class I (6.6%) and class IV (4.2%). In addition, it is possible to see the high comorbid conditions.

**Table 1:** Summary of baseline data.

Characteristic	Patients (n=903)
Mean age (years)	59.4±14.3
Male	482 (53.4%)
Female	421 (46.6%)
BMI (Kg/m^2^)	1.79±0.18
Heart failure	678 (75.1%)
NYHA class	
I	60 (6.6%)
II	284 (31.4%)
III	296 (32.7%)
IV	38 (4.2%)
EuroSCORE II	3.39±5.01 (0.5 - 58.6)
LVEF	60.6±12.0 (16 - 91)
Coronary artery disease	
Angina class IV	23 (2.5%)
Previous AMI	36 (4.0%)
Comorbid conditions	
Hypertension	551 (61.0%)
Renal impairment	56 (6.2%)
Renal replacement therapy	18 (2.0%)
Cerebrovascular disease	57 (6.3%)
COPD (moderated or severe)	49 (5.4%)
Active smoking	83 (9.2%)
Diabetes mellitus	163 (18.1%)
Cancer	24 (2.7%)
Endocarditis	74 (8.3%)
Antiplatelet therapy	66 (7.3%)
Assisted ventilation	17 (2.0%)
Use of the inotropic drug	183 (20.4%)
Intra-aortic balloon pump	2 (0.3%)
Pulmonary artery systolic pressure	43.6±17.0 (9 - 117)
Rhythm on ECG	
Sinus rhythm	771 (85.4%)
Arrhythmia	132 (14.6%)
Pacing	34 (3.7%)
Other	
PCI	29 (3.2%)
Percutaneous valvuloplasty	6 (0.66%)
Previous aortic valve implant	132 (14.6%)
Previous open-heart surgeries	173 (19.2%)
1 surgery	154 (17.0%)
2 surgeries	19 (2.1%)
ΔP (mmHg)	
Maximum ΔP	67.0±19.15
Medium ΔP	41.8±18.8

Values are mean±standard deviation or n (%)

AMI=acute myocardial infarction; BMI=body mass index; COPD=chronic obstructive pulmonary disease; ECG=electrocardiogram; Euro-SCORE=European System for Cardiac Operative Risk Evaluation; LVEF=left ventricular ejection fraction; NYHA=New York Heart Association; PCI=percutaneous coronary intervention

Table 2 summarizes the procedural information, a total of 903 patients underwent valve surgery, among them 455 isolated aortic valve replacement (AVR) (50.4%), 382 isolated mitral valve replacement (MVR) (42.3%), and 66 combined valve replacement or other intervention (7.3%). Subject follow-up compliance was detailed according to the visit interval: baseline (903), discharge (859), 3-6 months (859), and one year (846). Among the patients with indication for isolated AVR, 317 (69.6%) had aortic stenosis, 37 (8.1%) had aortic regurgitation, and 101 (22.2%) had a mixed diagnosis.

**Table 2:** Procedural details.

Procedural information	Patients (n=903)
Aortic valve replacement (AVR), isolated	455 (50.4%)
Aortic stenosis	317
Aortic regurgitation	37
Mixed	101
Mitral valve replacement (MVR), isolated	382 (42.3%)
Other interventions	66 (7.3%)
AVR + MVR	35
AVR + mitral valve plasty	6
AVR + tricuspid valve plasty	4
MVR + tricuspid valve plasty	17
AVR + MVR + tricuspid valve plasty	4
Surgical approach	@
Median sternotomy	882 (97.7%)
Hemisternotomy	21 (2.3%)
Implanted prosthesis	942
Aortic prosthesis	504
Mitral prosthesis	438

Values are n or n (%)

A total of 382 patients underwent isolated MVR, 35 with indication for AVR + MVR, 17 for MVR and tricuspid plasty, and four for AVR + MVR and tricuspid valve plasty, totaling 438 patients with MVR (isolated + combined).

Of the total surgeries, 13 (1.44%) were performed concurrently with aortic surgery and 47 (5.2%) were associated with coronary artery bypass grafting. The prevalent surgery approach was median sternotomy (97.7%). A total of 942 prostheses were implanted — AVR (n=504) and MVR (n=438). Supplementary materials S3 and S4 show the valve size distribution.

### Safety Data

Tables 3 and 4 show the key safety outcomes and AEs by position. Patients of the AVR group had no nonstructural valve dysfunction or structural valve deterioration throughout the study period.

**Table 3:** Summary of linearized complications rate after valve replacement with Bovine Pericardium Organic Valvular Bioprosthesis (BVP, Braile Biomédica®).

Complications	Aortic		Mitral	
	Early event rate^(a)^ (%)	Linearized late event rate^(b,c)^	Number of events/number of subjects	Linearized late event rate^(b,c)^
All-cause mortality	3.7	1.1	5.93	1.4
Stroke	1.97	0	0.91	0.45
All hemorrhage	3.5	0.45	3.41	0
All paravalvular leak	1.09	0	1.36	0
Structural valve deterioration	0	0	0.22	0
Nonstructural valve dysfunction (NSVD)^(d)^	0	0	0.91	0.45
Explant^(e)^	0.21	0	1.36	0.46
Reintervention^(e)^	0.21	0	1.13	0.46

(a) Early events include events that occurred on or before 30 days after the procedure

(b) Late events include events that occurred > 30 days after the procedure

(c) Late linearized rates (percent per patient-year) were calculated by dividing the number of late events by the sum of the late patient-years of experience and expressed as a percentage

(d) NSVD is inclusive of all paravalvular leak events; no NSVD of other etiology was observed

(e) One outcome was due to a procedure-related event and not a valve-related event

**Table 4:** Adverse events analysis after valve replacement with Bovine Pericardium Organic Valvular Bioprosthesis (BVP, Braile Biomédica®).

Adverse event	AVR (2 × OPC)^(c)^	AVR^(a,b)^ (% per patient-year)	MVR (2 × OPC)^(c)^	MVR^(a,b)^ (% per patient-year)
Thromboembolism	3.0	0.0	2.6	0.0
Valve thrombosis	0.08	0.22	0.06	0.0
Major hemorrhage	1.2	0.22	1.4	0.0
Major paravalvular leak	0.6	0.0	0.4	0.0
Endocarditis	1.0	0.68	0.8	0.72

AVR=aortic valve replacement; LPY=late patient-year; MVR=mitral valve replacement; OPC=objective performance criteria

(a) Late linearized event rate calculated by the number of events/LPY expressed as a percentage

(b) LPY is calculated from post-implant day 31 until the last day of contact

(c) OPC for tissue valves, as described in Table J.1 of EN-International Organization for Standardization 5840-2:2015, Annex J^[7]^

A total of 22 deaths were identified among the 455 patients of the AVR group included in the study. Of the 22 deaths, 17 occurred within 30 days after surgery (S5). Thirty-day mortality rates were 3.7% (AVR) and 5.9% (MVR), but among causes of death, 1.4% (n=13) was cardiac-related. For patients of the MVR group, one case of structural valve deterioration and four cases of nonstructural dysfunction were observed throughout the study period. There were 32 deaths among the 438 patients who underwent MVR included in this study. Of the 32 deaths, 26 occurred within 30 days after surgery (S5).

The most common cause of death considering the sum AVR + MVR was major infection/sepsis (n=11), followed by hemorrhage (n=8), non-identified (n=8), stroke (n=6), and renal failure (n=3).

According to linearized event rates (Table 3), the linearized late mortality rates were 1.1% (AVR) and 1.4% (MVR); stroke rates were 1.97% in the AVR group and 1.36% in the MVR group. All hemorrhage rate was 3.95% in the AVR group and 3.41% in the MVR group. All observed bleeding was related to anticoagulant use, and no case of hemolysis was caused by a paravalvular leak. Paravalvular leak rates were 1.09% in the AVR group and 1.36% in the MVR group. Structural valve deterioration rate was 0.22%. Nonstructural valve dysfunction was observed at an early rate of 0.91% of patients, or just one patient who underwent MVR.

Prosthetic valve endocarditis rate was 0.28%. All cases of valve endocarditis were observed in AVR patients, with native valve endocarditis caused by the same microorganism. These patients were all successfully reoperated. OPC are presented in Table 4.

### Objective Performance Criteria

Table 4 compares the late linearized rates for valve-related AEs to OPC (late complications > 30 days after implant surgery). As shown, in the results of comparative analysis with ISO 5840-2 OPC for AVR and MVR, all major AE rates were < 2 × OPC, just valve thrombosis in AVR is above the recommended value. However, only one case (1/438) occurred during the one-year follow-up and it was an isolated situation.

### Effectiveness

**Fig. 1:**
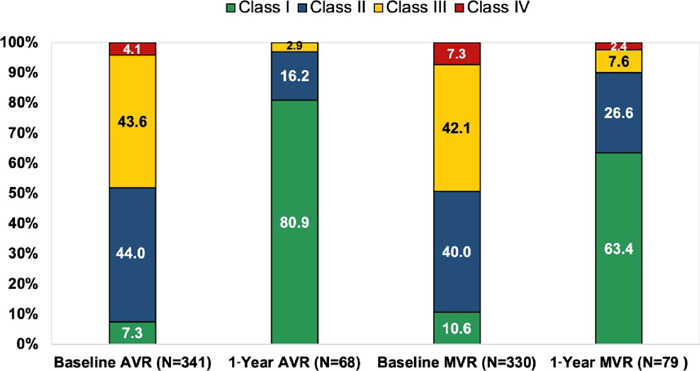
New York Heart Association classification of pre-procedure vs. post-procedure patients for different positions. AVR=aortic valve replacement; MVR=mitral valve replacement.

Analysis of effectiveness was based on the 903 patients that received BVP for 839 total patient-years. Figure 1 shows the patients’ NYHA functional classification at two time points: preoperative and 12-month follow-up for both positions (aortic and mitral). The patients included in these analyses have both preoperative and postoperative NYHA classification reported. The summary of changes in NYHA classification from baseline to one year for both MVR and AVR is given in S6. Patients also were classified according to heart failure (HF) for different positions: HF-AVR (baseline = 341 [74.9%] and one year = 68 [15.7%]), HF-MVR (baseline = 330 [75.3%] and one year = 78 [19.2%]), AVR without HF (baseline = 114 [25.1%] and one year = 365 [84.3%]), and MVR without HF (baseline = 108 [24.7%] and one year = 328 [80.8%]).

**Fig. 2:**
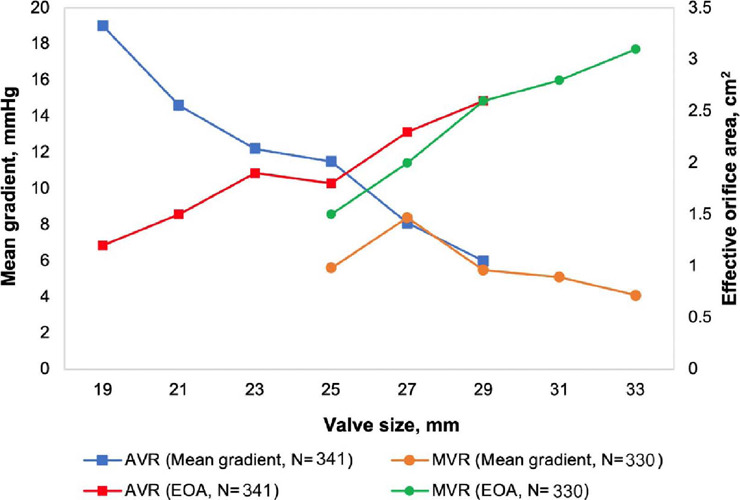
Hemodynamic results at one-year follow-up. AVR=aortic valve replacement; EOA=effective orifice area; MVR=mitral valve replacement.

Figure 2 summarizes temporal trends of key prosthesis hemodynamics (mean gradient and effective orifice area [EOA]) by different prosthesis sizes and implant positions at one-year follow-up. After surgery, hemodynamic performance was satisfactory in AVR and MVR patients, an effect that was maintained at 30 days and up to 12 months after surgical procedure for subjects with data available. Furthermore, the treatment with this biological heart valve shows low regurgitation index for AVR (none = 93.5%, mild = 5.8%, moderate = 0.5%, and severe = 0.2%) and MVR (none = 95.8%, mild = 2.1%, and moderate = 2.1%), as shown in S7.

### Survival and Freedom from Complications

**Fig. 3:**
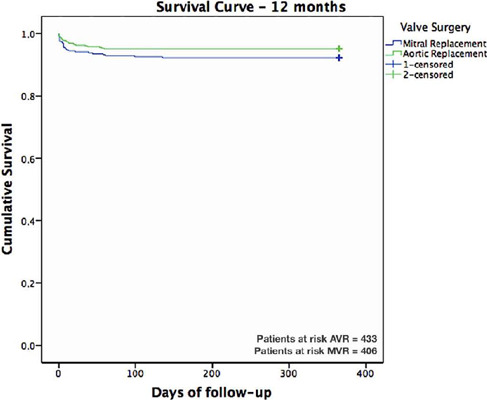
Survival curve stratified by valve surgery. AVR=aortic valve replacement; MVR=mitral valve replacement.

**Fig. 4:**
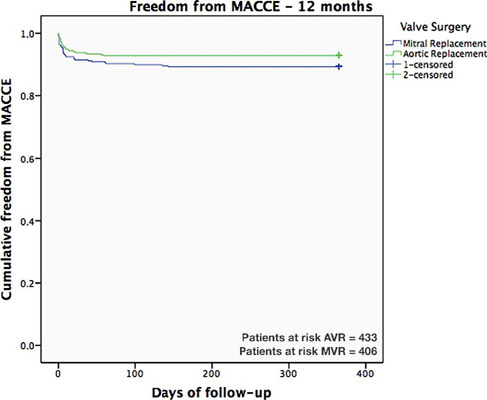
Major adverse cardiovascular and cerebrovascular event (MACCE)-free rate in aortic valve replacement (AVR) and mitral valve replacement (MVR) patients.

As shown in Figure 3, the survival curves for AVR and MVR after valve surgery were 95.1% and 92.7%, respectively. Figure 4 shows the comparative analysis of the one-year major adverse cardiovascular and cerebrovascular event (MACCE) event-free rate after valve surgery. As shown, the AVR MACCE event-free rate was 93.0% and the MVR MACCE event-free rate was 89.4%.

## DISCUSSION

According to standards, the ideal sample size is 800 patient-years. A minimum of 400 patient-years are required for each valve position for complete comparisons to OPC^[7^,^9]^. Clinical results of the BIOPRO Trial showed total compliance for all events described in the standard ISO 5840-2^[7]^, considering the BVP (Braile Biomédica®) results in both positions (aortic and mitral).

The results of this clinical retrospective study (BIOPRO) demonstrated safety with a low early mortality rate (< 30 days after implant) for AVR and a slightly higher rate for MVR. As a possible explanation, the slightly higher rate of early mortality rate can be influenced by several factors, mainly multiple valve replacements, associated procedures, and severe lesions associated with higher subject’s comorbidities, etc^[11,12]^.

As far as we observed, the prosthesis did not influence early mortality in the study population, among causes of death just 1.0% was cardiac-related. Demographic results demonstrated a high prevalence of several comorbidities, and procedure details showed a high occurrence of other valve interventions and surgeries associated with coronary artery bypass grafting. In addition, there is a known high prevalence of rheumatic valve disease, which is a major cause of heart disease in developing countries^[13]^. It was observed low late mortality (> 30 days after implant) similar to those described in the literature for other types of actual market bioprosthesis^[14,15,16]^.

When considered valve-related AE rates for the major safety endpoints (thromboembolism, valve thrombosis, major hemorrhage, major paravalvular leak, and endocarditis) were < 2 × OPC for a bioprosthetic valve, there was just one exception for valve thrombosis rate. However, only one case occurred during the period of > 30 days up to one year and it is an isolated situation. All data demonstrated an intrinsic safety of BVP that its intended use for AVR or MVR.

In the BIOPRO Trial, analysis of effectiveness is based on NYHA functional classification and echocardiographic hemodynamic data at one-year follow-up. There was an improvement in NYHA classification by at least one class for AVR and MVR patients at one year from baseline. AVR (93.5%) and MVR (95.5%) patients had no valvar regurgitation at one-year follow-up. In this context, based on hemodynamic performance, BVP demonstrated acceptable effectiveness considering its intended use to AVR or MVR, significantly promoting the improvement of hemodynamic performance and NYHA functional classification compared to baseline values.

Furthermore, echocardiographic evaluations were reevaluated by the independent Core Lab to assess performance of the device in terms of hemodynamic behavior based on control results of EOAs and mean gradients.

The benefit associated with BVP is also supported by results of the actuarial global survival rate and freedom of AE rate at the end of 12 months. Rate of freedom from all-cause mortality at one year was 93.9%, and the Kaplan–Meier overall survival curves indicated that freedom from all-cause mortality was 95.1% for AVR and 92.7% for MVR.

Three other market bioprosthetic heart valves had similar freedom from all-cause mortality rates — Magna Ease™ – Edwards (90.6%), Trifecta™ – St. Jude Medical (95.8%), and Avalus™ – Medtronic (96.4%)^[15,16,17]^. Another clinical trial that evaluated a randomized study comparing surgical *vs.* transcatheter AVR found that one-year all-cause mortality rate in the surgical AVR group was 7.5% (Kaplan– Meier estimate)^[18]^.

In addition, the BVP event-free rate was 90.7%; when related to the type of surgery they were 93.0% (AVR MACCE) and 89.4% (MVR MACCE). In a comparative analysis of late outcomes between the Trifecta™ and Magna Ease™ biological heart valves, the freedom from one-year MACCE in the Trifecta™ group was 93.9%, and in the Magna Ease™ group it was 94.1%^[19]^. This analysis indicated that the risks of BVP are similar to those observed with other surgical bioprosthetics in the market.

### Limitations

Due to the study’s retrospective design, there was a limitation in the number of exams evaluated by Core Lab; however, the number was considered significant (approximately 80%). Another limitation is that a control group was not included. It is tough to include a “gold standard” control group in studies of AVR and MVR, since market valves have particularities and limitations, thus, the best alternative was to use OPC recommended by Annex J (ISO 5840-2), which defines the reference standard for surgically implanted heart valve substitutes^[7]^.

## CONCLUSION

The BIOPRO Trial results analysis demonstrated excellent safety and clinical effectiveness of the BVP (Braile Biomédica®) for clinical application to heart valve replacement of malfunctioning natural or previously placed prosthetic valves when used under the indications for use, even in comparison to other commercial bioprosthetic heart valves available. The data support that BVP benefits outweigh the probable risks for AVR and/or MVR. This bioprosthesis performed well in aortic and mitral positions, considering that the late linearized rates were < 2 × OPC for all parameters, with low early and late mortality rates, as well as improvement in the patients’ NYHA classes.

## Figures and Tables

**S3:**
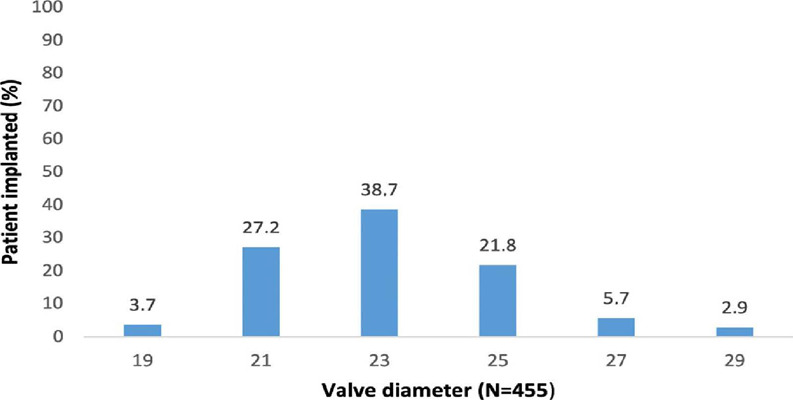
Distribution of valve sizes implanted in the aortic position.

**S4:**
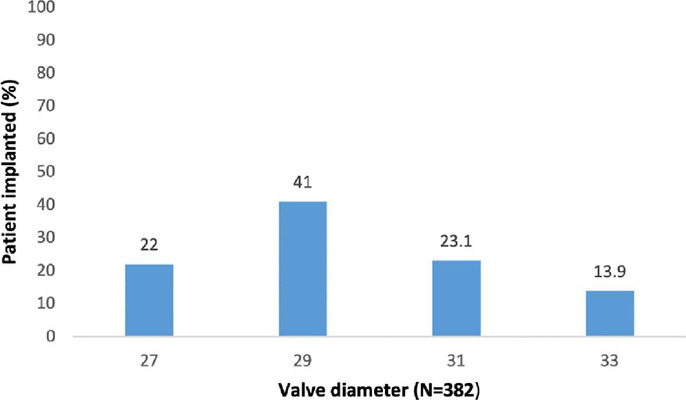
Distribution of valve sizes implanted in the mitral position.

**S6:**
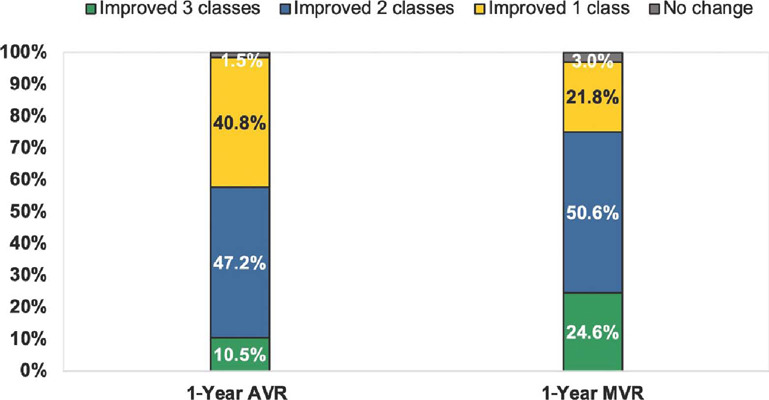
Change in New York Heart Association classification from baseline.

AVR=aortic valve replacement; MVR=mitral valve replacement

**S7:**
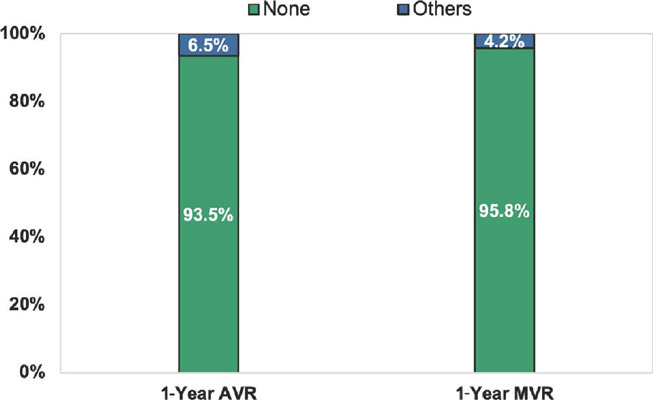
Valve regurgitation in 1-year follow-up.

AVR=aortic valve replacement; MVR=mitral valve replacement

## Abbreviations, Acronyms & Symbols

AEs: Adverse events
AMI: Acute myocardial infarction
AVR: Aortic valve replacement
BMI: Body mass index
BVP: Bovine Pericardium Organic Valvular Bioprosthesis
CABG: Coronary artery bypass grafting
COPD: Chronic obstructive pulmonary disease
ECG: Electrocardiogram
e-CRFs: Electronic Case Report Forms
EOA: Effective orifice area
EuroSCORE: European System for Cardiac Operative Risk Evaluation
HF: Heart failure
ISO: International Organization for Standardization
LPY: Late patient-year
LV: Left ventricular
LVEF: Left ventricular ejection fraction
MACCE: Major adverse cardiovascular and cerebrovascular event
MVR: Mitral valve replacement
NYHA: New York Heart Association
OPC: Objective performance criteria
PCI: Percutaneous coronary intervention

## Supplementary Material

**S1 Bovine Pericardium Organic Valvular Bioprosthesis**

**S2 Inclusion and Exclusion Criteria**

Patients who have already undergone aortic or mitral valve replacement and have been followed up in the selected institutions for one year were included.

**Inclusion Criteria (Group I – Aortic):**

- Symptomatic patients with severe aortic insufficiency.
- Asymptomatic patients with severe aortic insufficiency and with left ventricular ejection fraction (LVEF) at rest ≤ 50%.
- Patients with severe aortic insufficiency and undergoing coronary artery bypass grafting (CABG) or surgery of the ascending aorta or other valve.
- Asymptomatic patients with severe aortic insufficiency and with resting ejection fraction > 50% with severe left ventricular (LV) dilation: LV end-diastolic diameter > 70 mm or LVEF > 50 mm (or LVEF > 25 mm/m2 of body surface, in patients with small body size).
- Symptomatic patients with severe aortic stenosis of high gradient (mean gradient ≥ 40 mmHg or peak speed ≥ 4.0 m/s).
- Symptomatic patients with severe low flow low gradient (< 40 mmHg), aortic stenosis with reduced ejection fraction, and evidence of flow reserve (contractile) excluding pseudosevere aortic stenosis.
- Symptomatic patients with low flow aortic stenosis and low gradient (< 40 mmHg) with normal ejection fraction after careful confirmation of severe aortic stenosis.
- Symptomatic patients with low flow and low gradient aortic stenosis and reduced ejection fraction without flow reserve (contractile), particularly when the amount of calcium on computed tomography confirms severe aortic stenosis.
- Patients with symptomatic aortic stenosis with low surgical risk (Society of Thoracic Surgeons or European System for Cardiac Operative Risk Evaluation [EuroSCORE] II < 4% or EuroSCORE I logistical < 10% and no other risk factors not included in these scores, such as frailty, porcelain aorta, chest radiation sequelae).
- Asymptomatic patients with severe aortic stenosis and LV systolic dysfunction (LVEF < 50%) not due to another cause.
- Asymptomatic patients with severe aortic stenosis and an abnormal exercise test showing exercise symptoms clearly related to aortic stenosis.
- Asymptomatic patients with severe aortic stenosis and an abnormal exercise test showing a decrease in blood pressure below the baseline.
- Asymptomatic patients with normal ejection fraction and no abnormality of the exercise test, if the surgical risk is low and there is very severe aortic stenosis defined by a peak transvalvular velocity (Vmax) > 5.5 m/s.
- Asymptomatic patients with normal ejection fraction and no abnormality of the exercise test, if the surgical risk is low and severe valve calcification and Vmax progression rate ≥ 0.3 m/s/year.
- Asymptomatic patients with normal ejection fraction and no abnormality of the exercise test, if the surgical risk is low and there are high levels of type B natriuretic peptide markers.
- Asymptomatic patients with normal ejection fraction and no changes in exercise test, if the surgical risk is low and there is severe pulmonary hypertension (systolic arterial pressure of the resting pulmonary artery > 60 mmHg confirmed by invasive measure) without further explanation.
- Patients with severe aortic stenosis undergoing CABG or surgery of the ascending aorta or another valve.
- Patients with moderate aortic stenosis undergoing CABG or surgery of the ascending aorta or another valve after decision of the Heart Team.

**Inclusion Criteria (Group II – Mitral):**

- Symptomatic patients with severe primary mitral regurgitation and LVEF > 30%.
- Asymptomatic patients with severe primary mitral regurgitation and LV dysfunction (LVEF > 45 mm and/or LVEF < 60%).
- Asymptomatic patients with severe primary mitral regurgitation and preserved LV function (LVEF < 45 mm and LVEF > 60%) and atrial fibrillation secondary to mitral regurgitation or pulmonary hypertension (resting systolic pulmonary pressure > 50 mmHg).
- Asymptomatic patients with severe primary mitral regurgitation and preserved LVEF (> 60%) and LVEF 40-44 mm, with leaflet failure.
- Asymptomatic patients with severe primary mitral regurgitation and preserved LVEF (> 60%) and LVEF 40-44 mm, and presence of significant left atrial dilation (volume index ≥ 60 mL/m^2^ of body surface) in the sinus rhythm.
- Patients with severe primary mitral regurgitation and severe LV dysfunction (LVEF < 30% and/or LVEF > 55 mm) refractory to medical therapy.
- Patients with severe secondary chronic mitral regurgitation undergoing CABG and LVEF > 30%.
- Symptomatic patients with severe secondary mitral regurgitation, LVEF < 30%, but with the option for revascularization and evidence of myocardial viability.
- Patients with severe secondary mitral regurgitation and LVEF > 30% who remain symptomatic despite the ideal clinical treatment and with low surgical risk.
- Symptomatic patients with mitral stenosis (valve area ≤ 1.5 cm^2^) that are not suitable for percutaneous mitral commissurotomy.

**Exclusion Criteria:**

- Emergency surgical valve replacement.
- Aortic root surgical replacement.
- Patients who did not return for follow-up exams.
- Patients with renal failure, as determined by creatinine level ≥ 2.5 mg/dL or end-stage renal disease that requires chronic dialysis.
- Patients with stroke or transient ischemic attack within six months (180 days) before planned valve surgery.
- Patients with acute myocardial infarction within 30 days before planned valve surgery.
- Patients with any known life-threatening non-heart disease that will limit their life expectancy below one year.
- Patients diagnosed with abnormal calcium metabolism and hyperparathyroidism.
- LVEF ≤ 20%, as validated by diagnostic procedure before planned valve surgery.
- Echocardiographic evidence of intra-cardiac mass, thrombus, or vegetation.
- Hemodynamic or respiratory instability that requires inotropic support, mechanical circulatory support, or mechanical ventilation within 30 days before planned valve surgery.
- Documented leukopenia (leukocytes < 3.5×10³/µL), acute anemia (Hgb < 10.0 gm/dL or 6 mmol/L), or thrombocytopenia (platelet count < 50×10³/µL) accompanied by a history of hemorrhagic diathesis and coagulopathy.
- Patients who underwent organ transplantation.
- Pregnant or breastfeeding patients.
- Patients with a documented history of substance abuse (drugs or alcohol) in the last year before implantation.
- Concomitant positioning of the LV assist device.

**S5:** Causes of 30-day mortality.

Adverse events	AVR	MVR
Cardiac tamponade	0	2
Left ventricular failure	2	3
Hemorrhage	3	5
Acute renal failure	1	2
Major infection/sepsis	5	6
Stroke	3	3
Other	3	5
Total	17	26

AVR=aortic valve replacement; MVR=mitral valve replacement
